# FluoroGold-Labeled Organotypic Retinal Explant Culture for Neurotoxicity Screening Studies

**DOI:** 10.1155/2018/2487473

**Published:** 2018-02-13

**Authors:** Adrian Smedowski, Marita Pietrucha-Dutczak, Ruchi Maniar, Michael Ajeleti, Iwona Matuszek, Joanna Lewin-Kowalik

**Affiliations:** ^1^Chair and Department of Physiology, School of Medicine in Katowice, Medical University of Silesia, Medykow 18, 40-752 Katowice, Poland; ^2^Department of Ophthalmology, School of Medicine in Katowice, Medical University of Silesia, Ceglana 35, 40-514 Katowice, Poland

## Abstract

Preclinical toxicity screening of the new retinal compounds is an absolute requirement in the pathway of further drug development. Since retinal neuron cultivation and in vivo studies are relatively expensive and time consuming, we aimed to create a fast and reproducible ex vivo system for retinal toxicity screening. For this purpose, we used rat retinal explant culture that was retrogradely labeled with the FluoroGold before the isolation. Explants were exposed to a toxic concentration of gentamicin and ciliary neurotrophic factor (CNTF), a known neuroprotective agent. The measured outcomes showed the cell density in retinal ganglion cell layer (GCL) and the activity of lactate dehydrogenase (LDH) in the culture medium. Gentamicin-induced oxidative stress resulted in retinal cell damage and rapid LDH release to the culture medium (*p* < 0.05). Additional CNTF supplementation minimized the cell damage, and the increase of LDH release was insignificant when compared to LDH levels before gentamicin insult (*p* > 0.05). As well as this, the LDH activity was directly correlated with the cell count in GCL (*R* = −0.84, *p* < 0.00001), making a sensitive marker of retinal neuron damage. The FLOREC protocol could be considered as a fast, reproducible, and sensitive method to detect neurotoxicity in the screening studies of the retinal drugs.

## 1. Introduction

Retina and optic nerve diseases are one of the major causes of irreversible blindness worldwide, with increasing prevalence associated with aging of population [1]. Due to the development of a rapidly growing understanding of the pathomechanism of ocular disorders, novel ideas for their treatment and for drug delivery systems are being established [2]. The major reasonable route of retinal drug delivery is an intravitreal injection [2]. Although it provides the highest bioavailability of active compounds, the direct contact with the vitreoretinal compartment may result in interactions that can be either beneficial or toxic [3–5]. The beneficial success of these novel therapies cannot come at a price of safety or integrity of the tissue it is targeting; therefore, preclinical tolerability studies are performed as the first stage in the process of the evaluation of new drugs [6]. In case of the retina, the safety studies consider mostly *in vivo* intravitreal delivery of the active compound, and the histological evaluation of the retina remains the gold standard in retinal toxicity studies; however, some complementary methods examining the retinal morphology and function are also used [2]. For this purpose, rabbit, guinea pig, or rat models are the most commonly utilized as a basis for preclinical studies. In the case of bigger animals, that is, rabbits, ophthalmic examination, including funduscopy, fluorescein angiography, or optical coherence tomography can be performed [7–9]. However, there are growing evidences of applicability of optical coherence tomography in retina studies involving also small rodents [10–12]. In contrast to these methods, which evaluate only the retinal morphology, electrophysiological examination, that is, electroretinography, can provide information about the retinal function and integrity [2, 13–16]. The more advanced methods include tracking of delivered compounds with SPECT/CT cameras (single photon emission computed tomography), MALDI-MS (matrix-assisted laser desorption and ionization-mass spectrometry), or LC-MS/MS (liquid chromatography-tandem mass spectrometry) [17, 18]. Most of these methods require recruitment of highly specialized equipment, and the size of the small animals' eyeball (i.e., rodents) can be the limiting factor. As the demand for new pharmaceutical technologies in ocular therapies is high, there is a real need for creating economical and innovative, reproducible systems for the preclinical retinal drug toxicity screening that are equally efficient and rapid in terms of delivery.

Since there is no fully reliable and successful method of the retinal neuron culture (i.e., RGC), except complicated immunopanning separation method, and the *in vivo* studies are relatively cost and time consuming, the ex vivo organotypic retinal culture could be a competitive and highly efficient method for initial drug toxicity screening [14, 19–22]. The cultivation of ex vivo retinal tissue has major advantage over dissociated primary neuron culture, since in the whole tissue, the mutual multiple neuronal interactions and connections are still preserved, allowing to observe processes more closely mimicking those in living organism [23].

Initially, cultivation of neonatal retinal explants was reserved for studying retinal synaptic organizations, cell-cell interactions, axonal growth, and retinal cell differentiation using various culture settings [24–29]. The recent modification of retinal explant culture has been introduced by Johnson et al. In their model, retinal tissue isolated from adult rats is cultivated in system of culture inserts placed in wells containing culture medium [30, 31]. The semipermeable membrane that is forming the basis of insert isolates the explant from the culture medium and allows for selective passage of supplements added to the culture medium.

In this study, we propose a novel application of insert-cultured organotypic rat retinal explants, additionally prelabeled retrogradely by FluoroGold dye, as a fast and sensitive method, for safety studies of compounds delivered to the back of the eye.

## 2. Methods

### 2.1. Animals

**Figure 1:**
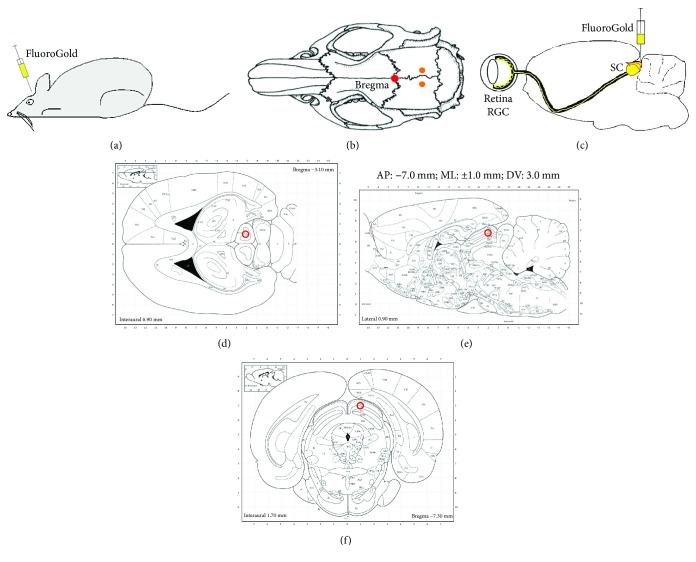
Stereotactic injection of the FluoroGold into optical portion of superior colliculi of the midbrain. (a–c) Principles of FG injection. (d–f) Horizontal, sagittal, and frontal section of rat brain with highlighted target of FG injection.

The study protocol has been approved by the Local Committee for an Animal Research and follows the ARVO statement for the use of Animals in Ophthalmic and Vision Research. In all experiments, we used approximately eight-week-old male Wistar rats weighing approximately 180 g (Center of Experimental Medicine, Medical University of Silesia, Katowice, Poland). For the retinal explant preparation, we used 20 animals. Twelve of them received a 3 *μ*l injection of 3% hydroxystilbamidine (FluoroGold, FG, Biotium, Fremont, CA, US) in 10% DMSO-saline into both the superior colliculi of the midbrain seven days before animals were sacrificed (FLOREC group). The specific contents of the injection allowed us to retrogradely label the retinal ganglion cells (RGC) [32]. The FG injection was performed under the general anesthesia with an intraperitoneal injection of ketamine (50 mg/kg; VetaKetam, Vetagro, Poland) and xylazine (5 mg/kg; Xylapan, Vetoquinol Biowet, Poland). The sites of injection were localized on the rat skull using stereotactic equipment. To ensure the correct localization of injection, the online atlas of the rat brain was used (Figure 1). Other eight animals were utilized without prior FG injection (OREC group).

### 2.2. Explant Preparation

**Figure 2:**
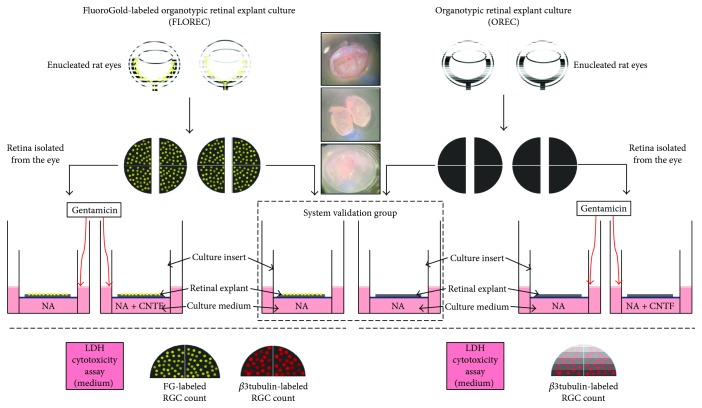
The layout of the experimental settings. The explants separated from 2 groups of Wistar rats—after FG injection (FLOREC) and without FG injection (OREC). System validation was used to determine whether the FG injection itself affects the retinal explant survival. Proper experiment consisted of comparable exposure of FLOREC and OREC explants to gentamicin and CNTF. The outcomes measured were RGC count for FG and *β*3tubulin and LDH activity in culture medium.

After seven days from the day of the initial FG injection, rats belonging to both study groups were sacrificed with an overdose of anesthetics (ketamine and xylazine) and subsequent decapitation. Immediately after euthanasia, the eyeballs were removed and collected in an ice-cold PBS solution containing 1% penicillin (Gibco, Carlsbad, CA, USA). After the anterior segments were removed, the retinas were isolated, cut into two halves through the vertical line, in the way that each explant contained half of superior and half of inferior retina and placed on culture inserts (0.4 *μ*m Millicell tissue culture insert, Millipore, Billerica, MA, USA) with the ganglion cell layer (GCL) upwards. During the isolation procedure, special care was taken to preserve the vitreous attached to the retinal surface. Inserts were placed in a twelve-well plate containing a culture medium consisting of Neurobasal A (NA, Gibco, Carlsbad, CA, USA) supplemented with 2% B-27 supplement (Gibco, Carlsbad, CA, USA), 1% N2 supplement (Invitrogen, Carlsbad, CA, USA), 1% penicillin solution (Gibco, Carlsbad, CA, USA), and 0.4% GlutaMax (Gibco, Carlsbad, CA, USA). Using the above-detailed method, we prepared 80 explants. The experiment was divided into three steps. For the first step (“*system validation group*”), we used 32 explants—16 FLOREC explants (previously labeled with FG) and 16 OREC explants (without FG labeling). These explants were cultured in standard medium (NA with standard supplementation as described above) for seven days at 37°C and 5% CO_2_. Every second day, the whole culture medium (500 *μ*l) was exchanged and the waste medium was collected for pH measurement and lactate dehydrogenase (LDH) cytotoxicity assay. After seven days, the explants were fixed in 4% paraformaldehyde (PFA) overnight at +4°C and then processed for immunostaining and GCL cell counting. In the second step of experiment (“*FLOREC/OREC comparative safety study*”), we cultured another 32 explants—16 FLOREC and 16 OREC explants. Eight of the explants from each group were cultured with standard supplemented NA medium with addition of 10 ng/ml of ciliary neurotrophic factor (CNTF, PeproTech, Rocky Hill, NJ, US), a neuroprotective agent. Four explants from each group were additionally exposed to a 1 *μ*g/ml concentration of gentamicin (G, Gibco, Carlsbad, CA, USA) supplemented to the condition medium on the fifth day of culture, a known inducer of oxidative stress, especially in neuron cell-expressing LDLR2 receptor. The selection of gentamicin as a neurotoxic agent is based on our previous observations involving retinal explant culture [33]. The explants were cultured for seven days at 37°C and 5% CO_2_. Every second day, the culture medium (500 *μ*l) was exchanged and the waste medium was collected for pH measurement and LDH cytotoxic assay. After seven days, the explants were fixed in 4% PFA overnight at +4°C and then processed for immunostaining and cell counting. In the third step of the experiment (*FLOREC safety study*), we used 16 FLOREC explants. Eight explants were cultured with standard supplemented NA medium with addition of 10 ng/ml CNTF. Four explants from each group (with and without CNTF) were exposed to 1 *μ*g/ml of gentamicin added to the condition medium on the third day of culture. The explants were cultured for seven days at 37°C and 5% CO_2_. Every second day, the culture medium (500 *μ*l) was exchanged and the waste medium was collected for LDH cytotoxic assay. After seven days, the explants were fixed in 4% PFA overnight at +4°C and then processed for immunostaining and GCL cell counting (Figure 2).

### 2.3. Immunostaining

After fixation, the floating samples were washed in the 0.05 M TBS overnight at +4°C and incubated in 20% NGS 0.1% Triton X-100 in 0.05 M TBS for 45 min. Primary antibody incubation was performed overnight at +4°C, after which samples were washed in 0.05 M TBS, pH 7.4 and incubated for 3 h at the room temperature (RT) with an appropriate secondary antibody and washed again. To counterstain nuclei, samples were incubated with 1 : 10,000 dilution of 4′,6-diamidino-2-phenylindole (DAPI, Sigma-Aldrich, St. Louis, MO, USA) for 10 min at RT and mounted with Mowiol (Calbiochem, San Diego, CA, USA). As a primary antibody, we used rabbit *β*3tubulin (dilution 1 : 300, Santa Cruz Biotechnology Inc, Santa Cruz, CA, US). As a secondary antibody, AlexaFluor 488 or 594 was applied (dilution 1 : 500, Thermo Fisher Scientific, Waltham, MA, US). Visualization was performed with a fluorescent microscope Zeiss Axio Scope.A1 (Zeiss, Oberkochen, Germany) and fluorescence microscope (Nikon, Japan). The RGC were counted manually using ImageJ software with Cell Counter plugin (http://imagej.nih.gov/ij/). For cell counting purpose, from each explant, photographs of six areas under 40x magnification for *β*3tubulin staining and FG labeling were obtained. Cell count is expressed as RGC density per mm^2^. Pictures were representing corresponding areas of peripheral (three pictures) and central (three pictures) retina. The cell count was expressed as a mean number of cells per visual field.

### 2.4. Colorimetric Cytotoxicity Assays

The release of LDH due to the cell membrane damage was detected by a CytoTox 96 nonradioactive cytotoxicity assay kit (Promega, Madison, WI, USA) according to the manufacturer's instructions. LDH activity in the culture medium was quantified using a plate reader (BIO-RAD Model 550, BIO-RAD, Hercules, CA, USA) with a measurement wavelength of 490 nm and a reference wavelength of 655 nm. Results were presented as optical densities (OD) or the OD ratio.

Since pH of the culture medium indirectly reflects the metabolic activity of retinal explants, we additionally measured the pH of waste medium.

### 2.5. Statistics

Statistical analysis was performed with the IBM statistical software SPSS 20 (IBM, Armonk, NY, USA). Descriptive statistical results were reported as a mean ± SD (standard deviation). Comparisons between groups were performed using an independent or a paired sample *t*-test. Multiple comparisons were performed using ANOVA test with post hoc Bonferroni correction. For the predictive relationship analysis, we used the Spearman correlation test. *p* values lower than 0.05 were considered statistically significant.

## 3. Results

### 3.1. Step 1: The System Validation Group

**Figure 3:**
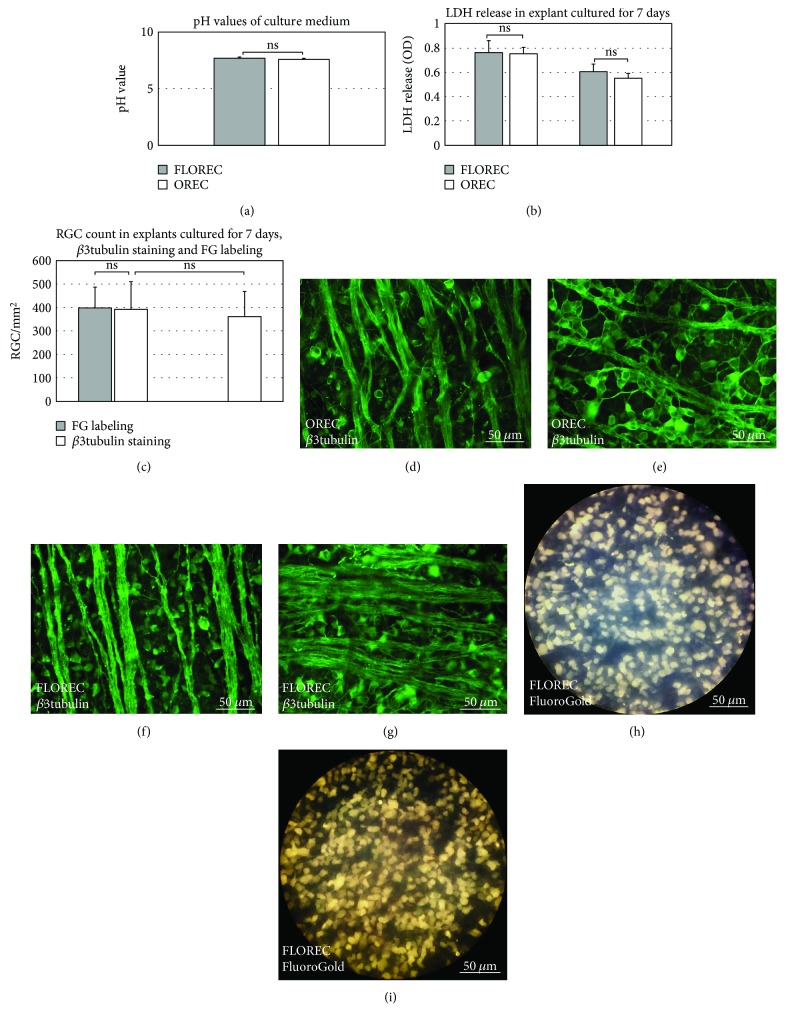
The system validation group. (a–c) Comparison of waste medium pH, medium LDH activity, LDH activity change between day 7 and day 3 (∆LDH), and RGC count for FG and *β*3tubulin in OREC and FLOREC explants after 7 days of culture. In FLOREC explants, the difference in number of cells labeled with FG and *β*3tubulin was not significant, similarly as the difference in number of *β*3tubulin cells in FLOREC and OREC explants. nFLOREC = 16 explants, nOREC = 16 explants. (d–g) Immunofluorescent staining of whole-mounted explants for *β*3tubulin after 7 days of culture. (h-i) FG-labeled whole-mounted FLOREC explants after 7 days of culture.

In this part of the experiment, we aimed to evaluate an impact of FG retrograde labeling of RGC on the quality of the retinal explants and to compare FLOREC and OREC explants for pH fluctuations of culture medium, LDH release into medium after seven days of culture, RGC survival for *β*3tubulin cell count, and correspondence of *β*3tubulin and FG labeling. The pH of FLOREC culture medium was 7.69 ± 0.08, and in OREC culture medium, it was 7.61 ± 0.02 (*p* = 0.2, *n* = 16/group, independent *t*-test, Figure 3(a)). The measured LDH release into culture medium of FLOREC explants on day seven was 0.76 ± 0.4 OD and in OREC medium 0.75 ± 0.2 OD (*p* = 0.9, *n* = 16/group, independent *t*-test, Figure 3(b)) and the LDH activity changes from day 3 to day 7 were also comparable. The mean RGC count in FLOREC explants for *β*3tubulin was 393 ± 117 cells/mm^2^ (469 ± 83 cells/mm^2^ in central and 318 ± 68 cells/mm^2^ in peripheral region), which was comparable with OREC explants—362 ± 106 cells/mm^2^ (437 ± 60 cells/mm^2^ in central and 286 ± 42 cells/mm^2^ in peripheral region; *p* = 0.5, *n* = 16/group, independent *t*-test). We did not find a statistical difference between the number of RGC stained with *β*3tubulin or FG in FLOREC explants (393 ± 117–469 ± 83 cells/mm^2^ in central and 318 ± 68 cells/mm^2^ in peripheral region and 399 ± 89–494 ± 94 cells/mm^2^ in central and 304 ± 81 cells/mm^2^ in peripheral region, respectively, *p* = 0.9, *n* = 16, paired *t*-test; Figures 3(c)–3(i)).

### 3.2. Step 2: FLOREC/OREC Comparative Safety Study

**Figure 4:**
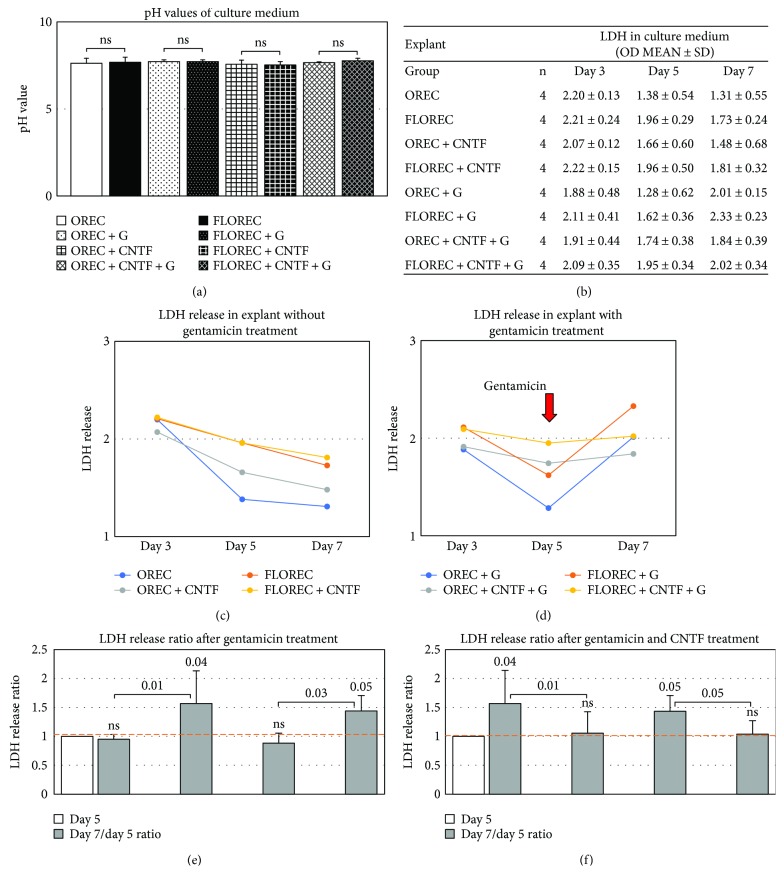
FLOREC/OREC comparative safety study. (a) The pH values of culture medium in OREC and FLOREC explants. (b) The absolute values of LDH activity in culture medium for FLOREC explants were slightly higher than for OREC explants without FG. (c-d) In explants without gentamicin insult (c), the LDH activity tended to decrease in time, while in case of explants after gentamicin exposure (d), there was rapid LDH outflux detected in culture medium due to cell membrane damage. The gentamicin insult was visibly alleviated by CNTF supplementation and resulted in almost 3-fold lower release of LDH. (e-f) Relative ratio comparisons of LDH activity. ANOVA *p* value for multiple comparisons = 0.0001. Bolded italic *p* values represent significance after post hoc Bonferroni correction.

After confirming that FG retrograde labeling of RGC does not negatively affect the FLOREC explants, we next aimed to compare the response of both types of explants to neuroprotective (i.e., CNTF) and neurotoxic (i.e., gentamicin) agents and the possibility of detecting induced fluctuations of LDH in the culture medium. In these settings, the pH of the culture medium ranged from 7.5 to 7.8 and the differences were insignificant (*p* > 0.05, *n* = 4 explants/group, independent *t*-test, Figure 4(a)). The absolute values of LDH activity in culture medium for FLOREC explants were slightly higher than for explants without FG; however, the difference was not significant (*p* > 0.05, *n* = 4 explants/group, independent *t*-test, Figure 4(b)). In relative comparison, the exposition of explants to gentamicin on the fifth day resulted in a rapid release of LDH into culture medium starting after this day of culture (Figures 4(c)-4(d)). In multiple comparisons, there was significant difference in LDH release in time (*p* = 0.0001, ANOVA). This calculated increase of LDH activity in culture medium of explants without CNTF treatment was 44% in FLOREC explants (*p* = 0.05, *n* = 4 explants/group, independent *t*-test) and 57% in OREC explants (*p* = 0.04, *n* = 4 explants/group, independent *t*-test) at day seven when compared to the fifth day of culture (Figure 4(e)). The CNTF treatment minimized the release of LDH from the retinal cells, reducing the overall increase of LDH release to 4% in FLOREC explants and 6% in OREC explants at day seven when compared to the fifth day of culture (*p* > 0.05, *n* = 4 explants/group, independent *t*-test, Figure 4(f)). In both types of cultured explants, gentamicin insult was related to significant release of LDH into culture medium when compared to untreated explants at the same time point, that is, day 7 (*p* = 0.01 in OREC and *p* = 0.03 in FLOREC explants, ANOVA with post hoc Bonferroni correction, Figure 4(e)). Additional CNTF treatment prevented gentamicin-induced LDH release when compared to explants that were not treated with CNTF at the same time point, that is, day 7 (*p* = 0.01 in OREC and *p* = 0.05 in FLOREC explants, ANOVA with post hoc Bonferroni correction, Figure 4(f)).

### 3.3. Step 3: FLOREC Safety Study

**Figure 5:**
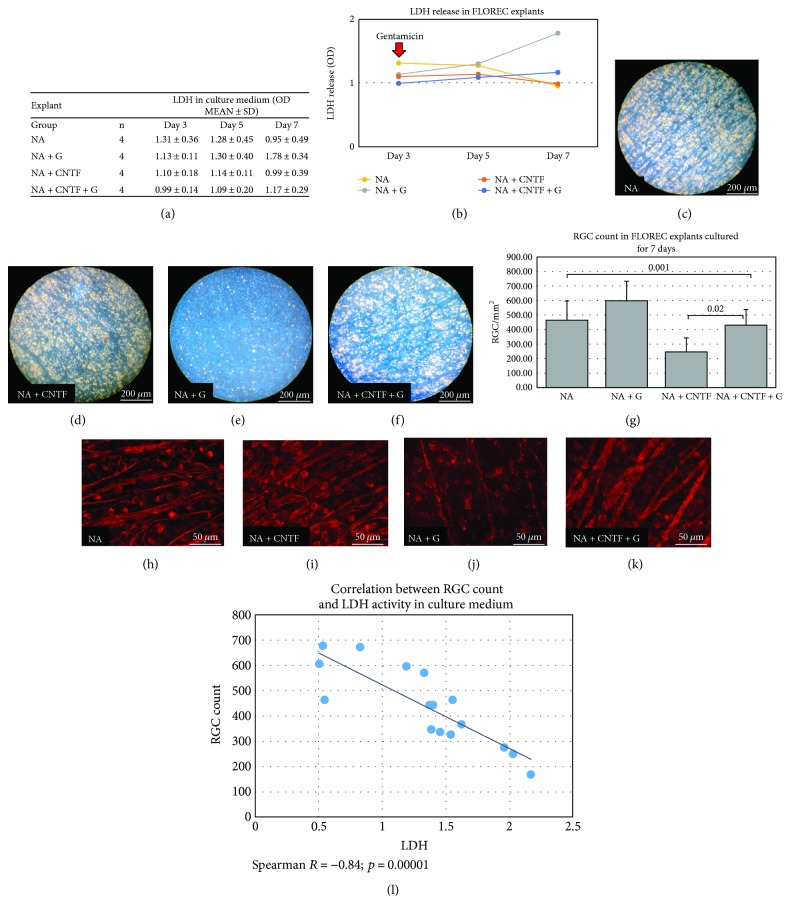
FLOREC safety study. (a-b) The absolute LDH activity in culture medium of FLOREC explants. The gentamicin insult introduced on the third day of culture resulted in accelerated release of LDH which was higher in explants without CNTF treatment. (c–f) FG-labeled whole-mounted explants representing study groups (after 7 days of culture). (g) RGC count in FLOREC explants after 7 days of culture. ANOVA *p* value for multiple comparisons = 0.001. Bolded italic *p* values represent significance after post hoc Bonferroni correction. (h–k) Immunofluorescent staining of whole-mounted explants for *β*3tubulin (after 7 days of culture). (l) The correlation between LDH activity in culture medium and RGC count after 7 days of explant culture.

After we demonstrated that both types of explant reactions are similar in either neurotoxic or neuroprotective environment, in the next stage, we performed a full screening study for CNTF and gentamicin using only FLOREC explants. The gentamicin insult introduced on day three resulted in an accelerated release of LDH by 9% in explants with additional CNTF treatment (*p* > 0.05, *n* = 4 explants/group, independent *t*-test), and 15% in explants without CNTF treatment (*p* > 0.05, *n* = 4 explants/group, independent *t*-test) on day five and by 17% (*p* > 0.05, *n* = 4 explants/group, independent *t*-test) and 57% (*p* = 0.01, *n* = 4 explants/group, independent t-test) on day seven, respectively. Explants cultured without gentamicin showed tendency for time-dependent decrease of LDH activity in the culture medium, as observed in the previous steps of experiment (Figures 5(a)-5(b)). These fluctuations of enzymatic activity in medium were linked with changes in RGC count for FG-labeling in GCL (*p* = 0.001, ANOVA test, Figures 5(c)–5(k)). CNTF treatment of gentamicin-induced explants resulted in higher RGC count when compared with group that was not treated with CNTF (430 ± 109 cells/mm^2^ versus 246 ± 96 cells/mm^2^, respectively, *p* = 0.02, ANOVA with post hoc Bonferroni correction, Figure 5(g)). In the correlation analysis, there was strong, significant negative relation between RGC count and LDH activity in culture medium (*R* = −0.84; *p* = 0.00001, *n* = 4/group, the Spearman test). The lower count of RGC, the higher activity of LDH in culture medium was observed (Figure 5(l)).

## 4. Discussion

In our current work, we present a new idea for the retinal drug toxicity screening method, based on organotypic ex vivo retinal explant culture. We have shown here that the retrograde labeling of the RGC with FluoroGold prior to the retinal explant isolation allows to simplify the quantitative evaluation of the retinal explants without affecting their survival. The new method we introduced includes analysis of the retinal cell membrane permeability for LDH as well as RGC quantification after the exposure to tested agents.

Attempts to create the protocol for the ex vivo retinal explant culture have been made since late 70s of the last century using goldfish models [34]. In 1981, Smalheiser et al. reported the first protocol for the fetal rodent (mouse) retinal organotypic culture [25]. Since then, the organotypic retinal cultures have become the method of growing interest as an independent setting for ocular research. Regardless of the utilized species—fish, mice, rats, rabbits, chickens, monkeys, pigs, bovines, or human, the ex vivo cultures were mostly used to observe processes associated with the degeneration of retinal neurons induced by denervation [24]. The 21st century brought significant development in retinal organotypic cultures and they started to be used as models, applied in neurodevelopmental studies as well as to seek opportunities to delay the neurodegenerative process or to replace the nonfunctional neurons [30, 31, 35–39]. Ex vivo retinal cultures have also become a substitute for disease models, that is, diabetic retinopathy or glaucoma [40, 41].

There are different approaches to prepare and maintain the retinal cultures, differing from the point of view of tissue separation, size of retinal pieces, and preferable culture medium [21, 23, 30, 38, 40–44]. In our study, we used a model based on rat retinal explants cultured in Neurobasal A medium described previously by Johnson et al., since it provides a good survival of retinal neurons after seven days [30, 45]. From our culture protocol, we excluded supplementation with streptomycin, because another aminoglycoside antibiotic (gentamicin) was applied as our tested agent. We also decided to divide retinas into 2 instead of 4 explants, to ensure more reliable representation of RGC, taking into account their asymmetric distribution in the retina. The retrograde labeling of RGC with fluorescent tracer FluoroGold is a widely used approach in eye research. Similarly, as it is shown in available *in vivo* data, and also in our ex vivo settings, the FG retrograde labeling does not seem to affect the RGC survival; however, FLOREC explants presented slightly higher initial LDH activity in culture medium which could be caused by DMSO used as a solvent for FG injection [32, 46–51]. Moreover, in our study, FG labeling did not impair the reactions of retinal explants to exposed treatment, which were comparable with those observed in unlabeled explants. Since the FG is a fluorescent dye, the only inconvenience that must be considered, special care during explant culture should be taken to avoid excess exposure of the explants to the light and to prevent fluorescence diminishing.

The cell membrane's permeability for LDH has been used widely as a method for cytotoxicity assays in different settings [52, 53]. In our study, the LDH membrane's permeability was affected by the toxic concentration of gentamicin and the protective mechanisms were activated using CNTF. Since LDH is released from the cells due to membrane damage, the initial absolute activity of this enzyme in culture medium varied due to explant preparation technique itself (the exact size of explants, time of isolation). In our reasoning, we made conclusions based on LDH activity changes after exposition to tested agents rather than on absolute values of LDH activity which could be affected by other external factors. It is known from neuro- and ototoxicity studies that aminoglycoside antibiotics can affect mitochondrial bioenergetics and induce reactive oxygen species (ROS) production in cells, which express the megalin receptor (LDLR2), like retinal neurons [33, 54, 55]. The energy failure related to aberrant mitochondrial metabolism and the ROS are affecting Bcl-2 gene, CNTF expression, activate stress kinase and the caspase family of proteases leading to subsequent neurodegeneration [56, 57]. The CNTF supplementation can alleviate the aminoglycoside-related insults, results in the overexpression of antiapoptotic Bcl-2 gene, reverses the aberrant mitochondrial bioenergetics, and reduces the ROS production [58]. Similarly, as in ototoxicity studies, in our experiment, we observed that CNTF treatment alleviated the toxicity of gentamicin in retinal explant culture. The LDH activity in culture medium appeared to be a sensitive marker of retinal cell damage (here correlated with the RGC count), which reflected the cytotoxic status of the whole retina, since gentamicin can damage also other than RGC populations of neurons [55]. In our study, the RGC cell damage (expressed in decreased cell count) was associated with an increased permeability of retinal cell membranes, leading to subsequent leakage of LDH that was then detected in the culture medium.

The screening method in its nature should be highly efficient, fast, repeatable, and low cost. The organotypic retinal explant culture as a model of neurodegeneration has been applied as a supplementary method for various studies in the area of the experimental retinal neuroprotection therapies. Undoubtedly, the basic advantage of this method is a short experiment duration—only seven days (twelve days, when considering the timing of the *in vivo* FG labeling), which can lower the costs of the experiment. Moreover, we should also pay attention to the ethical aspect: the retinal explant culture does not require utilization a large number of animals, as one retina can be divided into two explants. This fact also proves to be economical, but what is very important is that it allows us to study the effects of the selected agents at different concentrations on the retinal parts from the same animal in the same culture environment, excluding intraindividual variabilities. Although there are various protocols for retinal isolation and culture, their application (except the basic science studies), is limited. Here, we propose to consider the retinal explant culture assay as an applicable method in the testing of new ocular therapeutic agents. Perhaps, the application of these methods would allow the faster selection of effective drugs and reduce the time needed to test the drug in preclinical settings.

## 5. Conclusions

The FluoroGold-labeled organotypic rat retinal explant culture can be considered as a fast, reproducible, and sensitive method for safety studies of compounds delivered to the back of the eye.
